# Supplementary material for: Addressing the Endometriosis Knowledge Gap for Improved Clinical Care—A Cross-Sectional Pre- and Post-Educational-Intervention Study among Pakistani Women
Source: Healthcare (Basel). 2023 Mar 9;11(6):809. doi: 10.3390/healthcare11060809 (PMC10048359; doi:10.3390/healthcare11060809)
Supplement: Supplementary file 1 [file healthcare-11-00809-s001.zip › healthcare-2045668-supplementary.pdf]

Demographics

Q1. Age

- <18 years
- 18-29 years
- 30-39 years
- >40 years

Q2. Level of education

- Primary school
- Middle school
- High school
- Intermediate school
- Undergraduate
- Graduate

Q3. Healthcare worker

- Yes
- No

Q4. Access to annual healthcare

- Always
- Often
- Sometimes
- Rarely
- Never

Q5. Age at menarche

- <10 years
- 10-16 years
- >16 years

Q6. Duration of cycle

- <21 days
- 22-24 days
- 25-28 days
- 29-32 days
- 33-35 days
- >36 days
- Irregular

Awareness

Q7. Heard of endometriosis

- Yes
- No
- Not sure

Pre-intervention questions

Q8. Where does endometriosis originate from?

- Fallopian tubes
- Uterus
- Ovaries
- Vagina
- Don’t know

Q9. What are potential causes for pelvic pain (check any that apply)?

- Premenstrual syndrome
- Polycystic ovarian syndrome
- Ovulation
- Pelvic inflammatory disease
- Sexually transmitted infections
- Uterine fibroids
- Urinary tract infections
- Endometriosis
- Don’t know

Q10. Which of the following are symptoms of endometriosis?

- Irregular vaginal bleeding
- Heavy vaginal bleeding
- Pain with sexual intercourse
- Pain during periods
- Don’t know

Q11. What are the chances a woman with mild or moderate endometriosis will conceive?

- Very good
- Good
- Fair
- Poor
- Very poor
- Don’t know

Satisfaction of knowledge

Q12. Are you satisfied with the knowledge of symptoms related to your periods?

- Completely satisfied
- Somewhat satisfied
- Neither satisfied nor dissatisfied
- Somewhat dissatisfied
- Completely dissatisfied

Symptoms in the last 3 months

Q13. In the last 3 months, have you had pelvic pain with your periods?

- Always (3 or 3 periods)
- Often (2 of 3 periods)
- Occasionally (1 of 3 periods)
- Never

Q14. In the last 3 months, have you had pelvic pain in between one period cycle and the next?

- Always (3 of 3 periods)
- Often (2 of 3 periods)
- Occasionally (1 of 3 periods)
- Never

Q15. In the last 3 months, have you had pelvic pain while urinating or defecating during your period?

- Always (3 of 3 periods)
- Often (2 of 3 periods)
- Occasionally (1 of 3 periods)
- Never

Level of concern

Q16. Are you concerned about any symptoms related to your periods?

- Extremely concerned
- Moderately concerned
- Somewhat concerned
- Slightly concerned
- Not at all

Q17. Have you suspected your symptoms related to your periods are due to endometriosis?

- Yes
- No, but considered
- No, and not considered

Q18. If yes, did you discuss these symptoms with your doctor?

- Yes
- No, but considered
- No, and not considered

<u>Interventional brochure</u>

Please read the image carefully to educate yourselves about endometriosis:

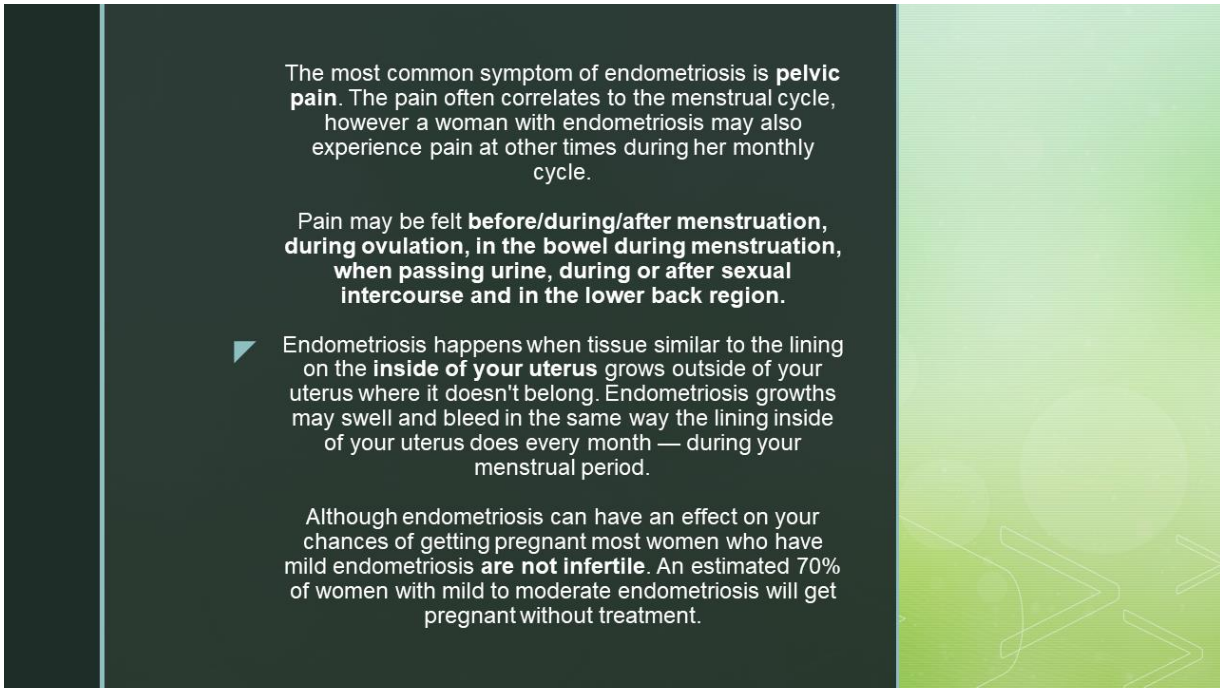

<u>Post intervention questions</u>

Q19. Where does endometriosis originate from?

- Fallopian tubes
- Uterus
- Ovaries
- Vagina
- Don’t know

Q20. What are potential causes for pelvic pain (check any that apply)?

- Premenstrual syndrome
- Polycystic ovarian syndrome
- Ovulation
- Pelvic inflammatory disease
- Sexually transmitted infections

- Uterine fibroids
- Urinary tract infections
- Endometriosis
- Don’t know

Q21. Which of the following are symptoms of endometriosis?

- Irregular vaginal bleeding
- Heavy vaginal bleeding
- Pain with sexual intercourse
- Pain during periods
- Don’t know

Q22. What are the chances a woman with mild or moderate endometriosis will conceive?

- Very good
- Good
- Fair
- Poor
- Very poor
- Don’t know
